# Interactions between amyloid, amyloid precursor protein, and mitochondria

**DOI:** 10.1042/BST20220518

**Published:** 2023-01-23

**Authors:** Heather M. Wilkins

**Affiliations:** 1 University of Kansas Alzheimer's Disease Center, Kansas City, KS, U.S.A.; 2 Department of Biochemistry and Molecular Biology, University of Kansas Medical Center, Kansas City, KS, U.S.A.; 3 Department of Neurology, University of Kansas Medical Center, Kansas City, KS, U.S.A.

**Keywords:** Alzheimer's disease, amyloid beta, amyloid precursor protein, bioenergetics, γ-secretase, mitochondria

## Abstract

Mitochondrial dysfunction and Aβ accumulation are hallmarks of Alzheimer's disease (AD). Decades of research describe a relationship between mitochondrial function and Aβ production. Amyloid precursor protein (APP), of which Aβ is generated from, is found within mitochondria. Studies suggest Aβ can be generated in mitochondria and imported into mitochondria. APP and Aβ alter mitochondrial function, while mitochondrial function alters Aβ production from APP. The role these interactions contribute to AD pathology and progression are unknown. Here, we discuss prior research, the rigor of those studies, and the critical knowledge gaps of relationships between APP, Aβ, and mitochondria.

## Introduction

**Figure 1.**
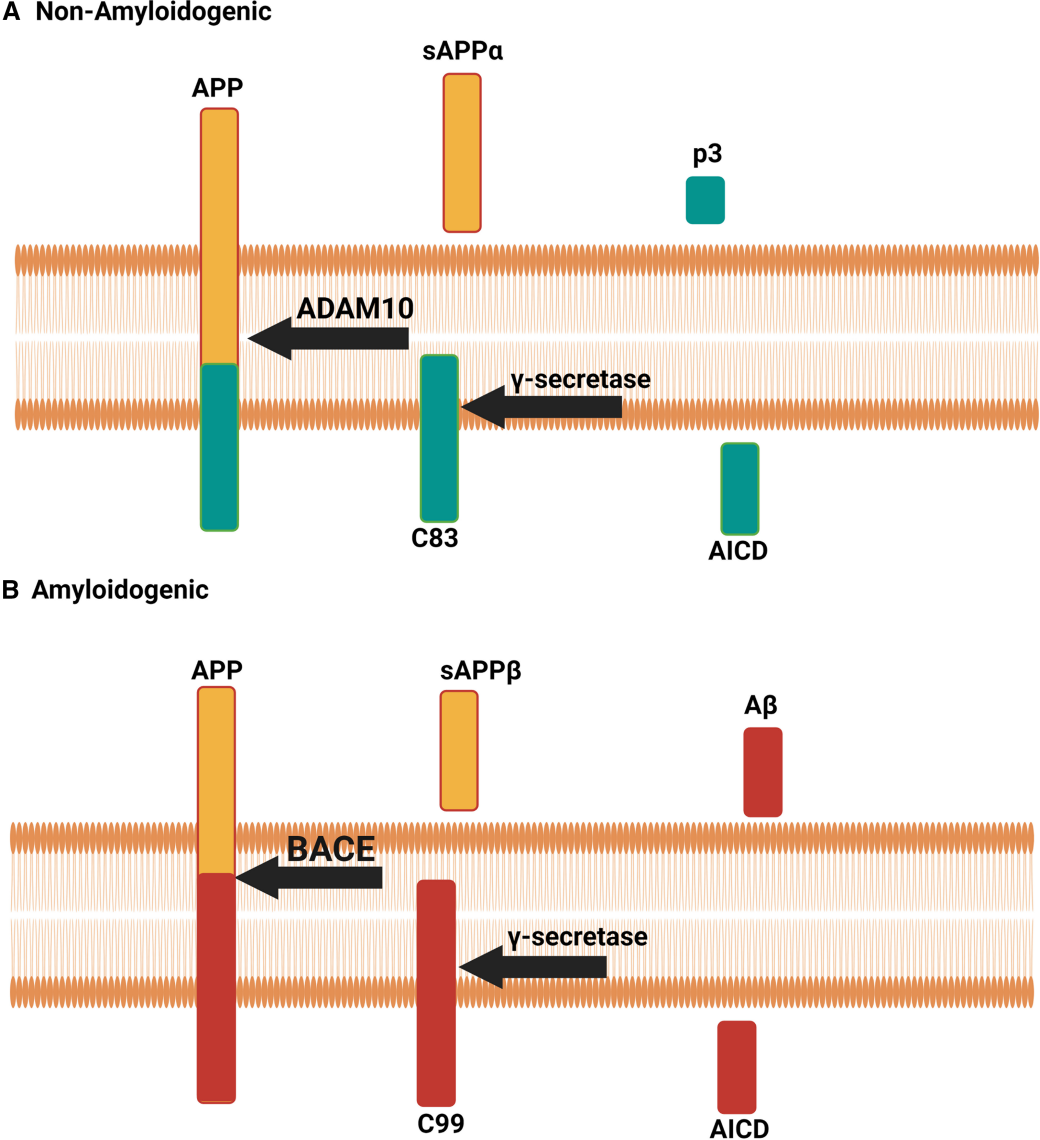
APP processing pathways. (**A**) Non-amyloidogenic APP processing. First, APP is cleaved by ADAM10 leading to the production of C83 and sAPPα. Next, C83 is cleaved γ-secretase leading to the production of AICD and p3. (**B**) Amyloidogenic processing. First, APP is cleaved by BACE1 leading to the production of C99 and sAPPβ. Next, C99 is cleaved by γ-secretase leading to the production of AICD and Aβ. Created using Biorender.

Amyloid beta (Aβ) plaques are a hallmark pathology of Alzheimer's disease (AD) and are a diagnostic requirement for AD [1,2]. Aβ is generated from cleavage of amyloid precursor protein (APP) by secretase enzymes [3]. Two APP processing pathways are currently known, the amyloidogenic and the non-amyloidogenic pathways (Figure 1). The non-amyloidogenic pathway is initiated with APP cleavage by α-secretase (ADAM10), generating soluble APPα (sAPPα) and C-terminal fragment 83 (C83) [4]. The γ-secretase enzyme complex cleaves C83 into p3 and the APP intracellular domain (AICD) [5]. In the amyloidogenic pathway, APP is first cleaved by β-secretase (BACE1), generating soluble APPβ (sAPPβ) and C-terminal fragment 99 (C99) [6]. The γ-secretase complex cleaves C99 generating Aβ and AICD. The γ-secretase complex is composed of presenilin 1 and or 2 (PS1, PS2-catalytic domain), anterior pharynx defective 1 (APH-1), presenilin enhancer 2 (Pen2), and Nicastrin. Cleavage by the γ-secretase complex occurs in a proof-reading manner, generating multiple Aβ species varying from 38 amino acids in length up to 42 amino acids [5]. Aβ_42_ is considered the most aggregation prone species.

Mutations in *APP* and *PS1/PS2* cause familial forms of AD [7]. Duplication of the *APP* gene, or chromosome 21 (in down syndrome) also lead to early onset AD [8]. Transgenic animals which express *APP* or *PS* mutations have Aβ aggregation within the brain without neuronal loss [9,10]. In many cases, cognitive changes occur in these transgenic models prior to Aβ aggregation/plaque formation which is the opposite of what occurs in human AD [11]. Overall, familial forms of AD account for <5% of all cases and data from transgenic models of familial AD do not recapitulate human disease. A major issue and critical knowledge gap is a lack of understanding of the function of APP and its processing/metabolism.

Research from the last several decades have described relationships between mitochondria, APP, Aβ, and γ-secretase. Numerous studies have established localization of APP, Aβ, and γ-secretase at or within mitochondria [12–20]. Conversely, association of APP and Aβ with mitochondria affects mitochondrial function and APP processing flux [3]. Here we will review the relationships between mitochondria, APP, Aβ, and APP processing.

## Relationships between APP processing and mitochondria

Both *in vitro* and *in vivo* studies describe a strong relationship between mitochondrial function and APP processing. The first studies examined the effects of sodium azide (cytochrome oxidase or COX inhibitor) and uncoupling of mitochondrial membrane potential using FCCP [21]. Gabuzda et al. treated COS cells with both sodium azide and FCCP and noted the accumulation of APP C99 and APP accumulation within the Golgi. COX inhibition alone also reduced sAPPα levels, suggesting decreased non-amyloidogenic APP processing. Bioenergetic stress through glucose deprivation and glycolysis inhibition decreased non-amyloidogenic APP processing [22]. Separate studies verified these findings in PC12 cells and noted that glycolysis inhibition decreased APP glycosylation, an effect that was mitigated through protein kinase c (PKC) [23]. Furthermore, glycolysis inhibition was found to effect APP trafficking to ER and Golgi in HEK cells [24].

Glycolysis flux and COX affect APP processing in numerous cell types. Fibroblasts from AD subjects reduced sAPPα production under conditions of glucose starvation [25]. These effects were enhanced with COX inhibition. Human fetal astrocytes showed increased Aβ production with glycolysis inhibition through both genetic knockdown of 6-phosphofructo-2-kinase/fructose-2,6-biphosphate (PFKB3) and pharmacological inhibition [26]. It is important to note that glycolysis and mitochondrial respiration are inversely linked, where decreased glycolysis can increase mitochondrial respiration. Furthermore, the downstream effects on reactive oxygen species (ROS), mitochondrial membrane potential, and ATP production were not measured in these studies. Overall, these initial studies depict a strong relationship between APP processing pathways and bioenergetic flux/mitochondrial function.

In transgenic AD mouse models altering mitochondrial function through changes to ROS decreases Aβ plaque burden. Reduced ROS production using two different methods led to a reduction in Aβ plaques with alterations in expression of secretase enzymes and full-length APP. The first study used overexpression of a mitochondrial targeted catalase in the Tg2576 AD mouse model, a method which targets H_2_O_2_ production [27]. The second study used overexpression of manganese superoxide dismutase (MnSOD, SOD2) in the Tg19959 AD mouse model, a method which targets superoxide production [28]. Overall, these studies suggest that altering redox and ROS influences APP processing and Aβ production.

Further *in vivo* studies have found that altering mitochondrial function through COX can also decrease Aβ production and plaque burden in AD transgenic mice. For example, reducing COX function through genetic knockdown of *COX10* in AD transgenic mice decreased Aβ plaque burden [29]. In human postmortem brain, higher COX Vmax was associated with higher Aβ burden in non-demented subjects [30]. These findings are interesting because COX Vmax and function are reduced in AD subjects both within the brain (postmortem studies) and systemically (fibroblasts, platelets) [31–34]. Overall *in vitro*, *in vivo*, and postmortem studies depict a strong relationship between COX function and Aβ production.

Alterations to complex I and III activity changes Aβ production both *in vitro* and *in vivo*. Human embryonic kidney (HEK) and SH-SY5Y cells treated with complex I (rotenone) and complex III (antimycin A) inhibitors increased both ROS and Aβ production [35]. In AD transgenic mice rotenone treatment or knockout of *Ndufs4,* a component of complex I, *also* increased Aβ levels. Further studies have shown that mild reduction in complex I activity reduces Aβ production and improves cognition in AD transgenic mice [36,37]. The mechanism of benefit for mild complex I inhibition is thought to occur through activation of AMP-activated protein kinase (AMPK) using a novel compound, CP2. CP2 also altered redox pairs, including NAD/NADH, AMP/ATP, and ADP/ATP [37]. Similar effects have been observed with metformin, an anti-diabetic drug that inhibits complex I and activates AMPK [38].

Mitochondrial DNA (mtDNA) influences APP processing and Aβ production. Mice which accumulate high levels of mtDNA mutations, showed increased Aβ plaque burden [39]. A separate study created congenic transgenic AD mice with varying mtDNA sequences through breeding [40]. This study created C57Bl/6 mice with mtDNA from other mouse strains including FVB/B, NOD/LtJ, and AKR/J. Altering the mtDNA sequences changed Aβ plaque burden and plaque size, while also altering mitochondrial function. Additional work targeted an endonuclease to the mitochondria, PstI in an AD transgenic mouse model. Expression of the mitochondrial targeted endonuclease reduced mtDNA copy number and reduced Aβ plaques and Aβ_42_ levels [41].

Understanding the role mtDNA may contribute to altered mitochondrial function and APP processing pathways requires a unique model. Cytoplasmic hybrids (cybrids) are cells which harbor a consistent nuclear DNA background with mtDNA from donors. Cybrids are created using cells which lack mtDNA through fusion with platelets from donors. Cybrids generated using platelets from AD subjects had increased ROS, decreased ATP, and decreased COX activity [42–46]. AD cybrids also had increased Aβ deposits and Aβ_40_/Aβ_42_ ratios. Overall, mtDNA could drive changes to mitochondrial function and alter APP processing pathways.

More recent studies have examined the role of specific mitochondrial changes and their relationship with APP processing. Using SH-SY5Y cells it was found that mitochondrial membrane potential altered Aβ secretion and APP localization to mitochondria [47]. Mitochondrial depolarization reduced Aβ secretion, but increased intracellular Aβ and mitochondrial localized APP. Hyperpolarization of mitochondria increased Aβ secretion, but decreased intracellular Aβ and mitochondrial localized APP. These results were verified using induced pluripotent stem cell (iPSC) derived neurons.

Data spanning decades across *in vitro*, *in vivo*, and postmortem studies describe a strong relationship between mitochondrial function and APP processing. Further research is needed to understand how this relationship may drive AD pathology and cognitive dysfunction. Additional studies have described localization of APP, Aβ, and γ-secretase at mitochondria. There is some equipoise regarding the localization of APP and γ-secretase at mitochondria and how this affects mitochondrial function. These studies and gaps in knowledge are discussed below.

## The effects of APP and APP processing on mitochondria

### Full length APP

APP localizes to mitochondria and evidence of this has been documented across numerous studies and models [12,15–17,47,48]. Localization of APP at mitochondria is noted in human cell culture, mouse models, and postmortem human brain samples. APP is associated with mitochondria and in mitochondrial associated membranes (MAMs) [14,49]. MAMs are contact sites between the ER and mitochondria which regulate mitochondrial function, calcium flux, and mitophagy.

APP is synthesized and glycosylated within the ER and its currently unknown if APP localization at mitochondria is a result of direct translocation from the ER or translocation from other cell membrane components, like the plasma membrane. Studies and evidence suggest that three positive amino acids in the N-terminus of APP do impact its localization to mitochondria [12]. When those amino acids are mutated to neutral charged amino acids this reduces APP mitochondrial localization. These findings are interesting and suggest APP localizes to mitochondria based on charge-charge interactions, given the overall negative charge of the mitochondrial matrix. More detailed studies are necessary to understand the mechanisms of APP translocation to the mitochondria.

APP localization at mitochondria alters mitochondrial function. *In vitro* experiments utilizing a wide variety of transformed cell types (PC12, HCN1A, SH-SY5Y, N2a cells) have shown that wild-type (WT) APP expression reduced bioenergetic function of cells, including reduced ATP production, COX activity, and altered calcium homeostasis [12,48,50,51]. Other studies have shown that loss of APP at mitochondria also reduces mitochondrial function, with reduced ATP production, increased ROS, and changes to mitochondrial membrane potential [52–54]. APP null mice further support that APP is important for mitochondrial function and cognition. APP knockout (KO) mice have reduced motor performance with increased astrogliosis [55,56]. Astrocytes isolated from APP KO mice have altered calcium homeostasis with reduced mitochondrial calcium and altered mitochondrial structure.

A highly cited study suggests APP interacts with TOM and TIM complexes [17]. The conclusion of the study was that this interaction between APP/TOM/TIM blocks import of nuclear encoded mitochondrial proteins. Using blue native gel electrophoresis high molecular weight complexes positive for APP, TOMM40, and TIMM23 were observed in mitochondrial fractions from postmortem human AD brain [17]. It should be noted that mitochondrial localized APP and these high molecular weight complexes were not observed in postmortem human brain tissue from normal control subjects. This finding is inconsistent with other studies, which depict APP localization at mitochondria in normal control human postmortem brain [57,58]. The AD postmortem brain mitochondria had reduced COX expression and reduced import of COX. This study did not directly show that APP blocks import channels of mitochondria and did not directly show that APP mitochondrial localization blocks import of nuclear encoded proteins into the mitochondria. This highlights a major knowledge gap in the field.

### Soluble APP

The association of sAPP species, either sAPPα or sAPPβ with mitochondria have not been established. However, through signaling mechanisms sAPPα indirectly modulates mitochondrial function. sAPPα exerts neurotropic effects through phosphatidylinositol-3-kinase-Akt kinase (PI3K) and p42/p44 mitogen-activated protein kinase (ERK1/ERK2) pathways [59]. In additional studies examining myotubes, PI3K mediated changes to glucose uptake through altered APP processing, including sAPPα levels [60]. Furthermore, sAPPα is protective against complex I inhibition through rotenone toxicity in mice expressing WT APP. The protective mechanism is likely mediated by PI3K signaling [61]. The overall neuroprotective effects of sAPPα have been reviewed elsewhere [62]. There is a lack of research examining the direct effects of sAPP species on mitochondria and vice versa. Fulfilling this knowledge gap is critical for the AD field.

### C-terminal fragments of APP

The role of APP versus APP fragments in mitigating effects on mitochondria is not clearly understood. Transgenic AD mouse models, including the 5X FAD model, accumulate C99 within mitochondria [16,58]. Accumulation of C99 in mitochondria resulted in reduced complex II function, increased DNA damage, and cytochrome c release from mitochondria. When BACE1 expression was reduced mitochondrial function was improved in the 5X FAD mouse model. In mouse embryonic fibroblasts (MEF) with PS knockout, C99 accumulates in MAMs and this leads to an overall increase in MAM content [20]. C99 has also been shown to accumulate in MAMs, and this was associated with decreased mitochondrial respiration and altered lipid content. Overall, the effects of C83 on mitochondria have not been investigated directly but is found within mitochondria [58].

### AICD

AICD is a 57 to 59 amino acid fragment generated from APP CTF upon γ-secretase cleavage. Studies show that AICD can translocate to the nucleus with its coactivator, Fe65 where they modulate gene transcription. Recently AICD has been found within mitochondria [58]. Increased expression of AICD and Fe65 reduce mitochondrial membrane potential, ATP production, and superoxide production [63]. The mechanism was linked to altered actin dynamics and downstream mitochondrial morphology/distribution changes. A separate study suggests that AICD alters PINK1 control of mitophagy and mitochondrial dynamics through altered forkhead box O3a (FOXO3a) activity [64]. Furthermore, AICD can inhibit the transcription of coiled-coil-helix-coiled-coil-helix domain containing 6 (CHCHD6). CHCHD6 regulates mitochondrial contact site and cristae organization [65]. When AICD is specifically targeted to mitochondria it induces apoptosis in a hippocampal cell line, and this effect could be rescued with antioxidants and caspase inhibitors [66]. The effects of AICD on mitochondria are likely due to its ability to change gene transcription in the nucleus and its localization within mitochondria. Its currently not known how AICD affects mitochondrial function, and whether it alters mitochondrial gene transcription.

### Amyloid beta (Aβ)

Aβ is also found within mitochondria across multiple model types including cell cultures, mouse models, and postmortem human brain. The origin of mitochondrial Aβ could be a consequence of local APP processing or through import into the mitochondria. Several studies report a functional γ-secretase complex within mitochondria capable of generating Aβ [13,15,19,67]. Thus far limited studies have reported BACE1 co-localization with mitochondria *in vitro* [68]. Other studies have shown that Aβ is directly imported into mitochondria [18].

The first study to show that Aβ was imported into mitochondria leveraged isolated rat brain mitochondria with exogenous Aβ [18]. Mitochondrial localized Aβ was also shown in human brain tissue using immunogold labeling with EM. The data from this study supports a role for TOM and TIM in the import of Aβ into mitochondria. Additional studies have shown Aβ import into mitochondria and subsequent degradation by the mitochondrial presequence peptidase (PreP) [69]. The interaction between Aβ and PreP can inhibit mitochondrial presequence processing of other mitochondrial proteins, leading to a reduction in mitochondrial peptide turnover in the matrix [70].

Aβ affects mitochondrial function *in silico*, *in vitro*, and *in vivo*. Incubation of isolated mitochondria with Aβ_42_ and copper lead to a dose dependent reduction in COX activity, this was associated with increased high-molecular weight Aβ_42_ species [71]. In AD transgenic J20 mice, Aβ is found within mitochondria and is associated with mitochondrial dysfunction at complex III and COX [72]. Aβ was shown to interact with Aβ-binding alcohol dehydrogenase (ABAD) in yeast two-hybrid screens, in transgenic AD mice (J20), and postmortem human brain. The interaction between ABAD and Aβ reduced nicotinamide adenine dinucleotide (NAD) binding and altered cognition in transgenic AD mice [73]. Primary neurons cultured from transgenic AD mice overexpressing ABAD had increased ROS production, reduced ATP, and reduced COX activity [74]. Using a separate AD transgenic mouse model with mutant APP and PS1, Aβ accumulation was associated with early mitochondrial dysfunction with increased mitophagy and autophagy [75]. Tg2576 AD mice and neuroblastoma cells expressing mutations in APP have Aβ localized to mitochondria [76]. These models also had increased H_2_O_2_ production which correlated with the levels of Aβ. It was further found that COX activity was reduced [76]. Accumulation of H_2_O_2_ and decreased COX activity were noted before Aβ plaque deposition. Further studies have shown that Aβ can disrupt mitochondrial fission and fusion dynamics *in vitro* especially through direct interactions with Drp1 [77,78].

Aβ can be generated in MAMs and oligomers of Aβ increase MAM content and mitochondrial calcium content [79]. An active γ-secretase localizes to MAMs and reducing γ-secretase expression/activity alters MAM number [80]. MAM numbers and function are increased in AD, which is hypothesized to increase Aβ production and alter mitochondrial function [49]. MAMs resemble lipid rafts which would facilitate APP processing pathways by secretase enzymes. Aβ can increase MAM number and alter calcium uptake into mitochondria [49,81]. Post-translational modifications of APP, such as lipidation by palmitoleic acid, can alter its localization to MAMs. Reductions in MAM content and activity was shown to reduce APP processing into Aβ, particularly palmitoylated APP within synapses [14].

The relationship between Aβ and mitochondrial function/MAMs has been a large focus of research within the AD field. Despite this, most studies have focused on models which overexpress or expose cells animal models to large amounts of exogenous Aβ. These types of models are difficult to draw firm conclusions from regarding the endogenous function of Aβ within mitochondria. Furthermore, additional studies are required to understand the role of mitochondrial function in Aβ production.

## Concluding remarks

**Figure 2.**
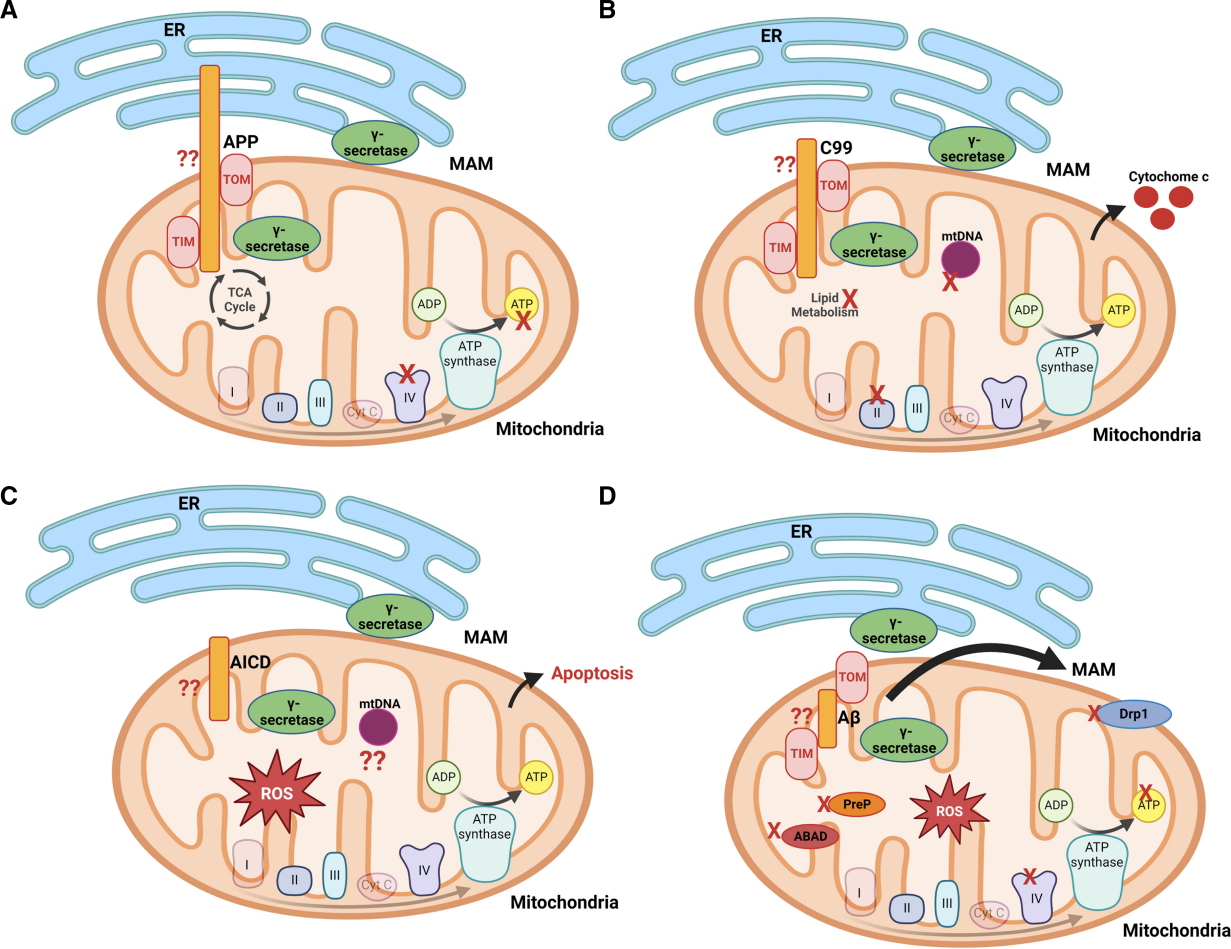
APP, APP fragments, and mitochondria. Current knowledge and knowledge gaps. (**A**) Full-length APP and mitochondria. It is unknown if APP translocates into the mitochondria from the ER through MAMs. It is also unknown if APP modulates MAM function. Studies suggest APP inhibits COX (complex IV) and ATP production. Some studies suggest APP associates with TOM and TIM. These findings have not been directly confirmed. Its also unclear the role of full-length APP versus APP fragments. (**B**) CTF of APP (C99) and mitochondria. It is unknown if C99 translocates into the mitochondria or is generated within mitochondria. Studies suggest C99 inhibits complex II, lipid metabolism, damages mtDNA, and induces cytochrome c release. The role of C99 has not been confirmed as independent from Aβ. (**C**) AICD and mitochondria. It is unknown if AICD translocates into the mitochondria or is generated within mitochondria. Studies suggest AICD induces ROS and apoptosis when it is localized to mitochondria. Its unknown how it affects mtDNA or transcription of mitochondrial genes. (**D**) Aβ and mitochondria. It is unknown if Aβ translocates into the mitochondria, is generated within mitochondria, or both. Studies suggest Aβ inhibits COX, ATP production, PreP, ABAD, Drp1, induces ROS, and increases MAM content. Created using Biorender.

This review focused on studies which examined the relationship between APP, APP processing, and mitochondria. The data supported by current models is outlined in Figure 2. Caution should be taken when drawing conclusions from these current studies as discussed below and highlighted in Figure 2.

Despite decades of research there is still no consensus on the function of APP, why APP is processed to fragments including Aβ, and what causes higher rates of Aβ production in AD. Mitochondrial dysfunction is highly prevalent across AD studies and models. Mitochondrial dysfunction has been shown to alter APP processing and Aβ production. Conversely, the production of Aβ (and other APP fragments) directly alter mitochondrial function. However, the role of full-length APP versus APP proteolytic fragments in altering mitochondrial function is unknown.

A functional γ-secretase is localized within mitochondria and full-length APP and its fragments are also found within mitochondria. Localization of APP, APP fragments, and γ-secretase to mitochondria is observed in both normal control and AD postmortem human tissue. However, one highly cited study depicted localization of APP within mitochondria only in AD human postmortem tissue. This study further suggested that APP blocks the import channels of mitochondria. Many conclusions have been made based on this single study which did not directly show that APP blocks mitochondrial protein import. It has been widely assumed that APP localization at mitochondria is ‘bad', but this is based on studies with APP overexpression models. Therefore, studies with endogenous APP protein expression are required to understand the role of APP and its fragments at mitochondria.

## Perspectives

- APP processing to Aβ and mitochondrial dysfunction are observed in AD subjects and experimental models. There are clear relationships between mitochondrial function, APP processing, and Aβ.
- Currently the field has focused on Aβ causing mitochondrial dysfunction, with studies suggesting APP inhibits protein import into mitochondria. Further evidence supports the processing of APP to Aβ within mitochondria.
- Future research should focus on directly assessing the function of APP and Aβ at mitochondria. The field should leverage models which do not overexpress or expose model systems to high levels of exogenous proteins.

## Abbreviations

ABAD: Aβ-binding alcohol dehydrogenase
AD: Alzheimer's disease
AICD: APP intracellular domain
AMPK: AMP-activated protein kinase
APP: amyloid precursor protein
CHCHD6: coiled-coil-helix-coiled-coil-helix domain containing 6
HEK: human embryonic kidney
KO: knockout
MAMs: mitochondrial associated membranes
NAD: nicotinamide adenine dinucleotide
PI3K: phosphatidylinositol-3-kinase-Akt kinase
ROS: reactive oxygen species
WT: wild-type

## References

[1] Braak, H. and Braak, E. (1997) Diagnostic criteria for neuropathologic assessment of Alzheimer's disease. Neurobiol. Aging 18, S85–S88 10.1016/s0197-4580(97)00062-6 9330992

[2] Committee NS. (2020) Coding Guidebook for the NACC Neuropathology Data Form. 11

[3] Wilkins, H.M. and Swerdlow, R.H. (2017) Amyloid precursor protein processing and bioenergetics. Brain Res. Bull. 133, 71–79 10.1016/j.brainresbull.2016.08.009 27545490 PMC5316384

[4] Allinson, T.M., Parkin, E.T., Turner, A.J. and Hooper, N.M. (2003) ADAMs family members as amyloid precursor protein alpha-secretases. J. Neurosci. Res. 74, 342–352 10.1002/jnr.10737 14598310

[5] Wolfe, M.S. and Miao, Y. (2022) Structure and mechanism of the gamma-secretase intramembrane protease complex. Curr. Opin. Struct. Biol. 74, 102373 10.1016/j.sbi.2022.102373 35461161 PMC9189058

[6] Cai, H., Wang, Y., McCarthy, D., Wen, H., Borchelt, D.R., Price, D.L. et al. (2001) BACE1 is the major beta-secretase for generation of Abeta peptides by neurons. Nat. Neurosci. 4, 233–234 10.1038/85064 11224536

[7] www.alzforum.org/mutations

[8] Salehi, A., Ashford, J.W. and Mufson, E.J. (2016) The link between Alzheimer's disease and down syndrome. A historical perspective. Curr. Alzheimer Res. 13, 2–6 10.2174/1567205012999151021102914 26487155 PMC6368451

[9] Uddin, M.S., Kabir, M.T., Rahman, M.S., Behl, T., Jeandet, P., Ashraf, G.M. et al. (2020) Revisiting the amyloid cascade hypothesis: from anti-abeta therapeutics to auspicious new ways for Alzheimer's disease. Int. J. Mol. Sci. 21, 5858 10.3390/ijms21165858 32824102 PMC7461598

[10] Hall, A.M. and Roberson, E.D. (2012) Mouse models of Alzheimer's disease. Brain Res. Bull. 88, 3–12 10.1016/j.brainresbull.2011.11.017 22142973 PMC3546481

[11] Jack CR, J., Barrio, J.R. and Kepe, V. (2013) Cerebral amyloid PET imaging in Alzheimer's disease. Acta Neuropathol. 126, 643–657 10.1007/s00401-013-1185-7 24100688 PMC3887147

[12] Anandatheerthavarada, H.K., Biswas, G., Robin, M.A. and Avadhani, N.G. (2003) Mitochondrial targeting and a novel transmembrane arrest of Alzheimer's amyloid precursor protein impairs mitochondrial function in neuronal cells. J. Cell Biol. 161, 41–54 10.1083/jcb.200207030 12695498 PMC2172865

[13] Behbahani, H., Pavlov, P.F., Wiehager, B., Nishimura, T., Winblad, B. and Ankarcrona, M. (2010) Association of Omi/HtrA2 with gamma-secretase in mitochondria. Neurochem. Int. 57, 668–675 10.1016/j.neuint.2010.08.004 20705111

[14] Bhattacharyya, R., Black, S.E., Lotlikar, M.S., Fenn, R.H., Jorfi, M., Kovacs, D.M. et al. (2021) Axonal generation of amyloid-beta from palmitoylated APP in mitochondria-associated endoplasmic reticulum membranes. Cell Rep. 35, 109134 10.1016/j.celrep.2021.109134 34010653 PMC8287518

[15] Del Prete, D., Suski, J.M., Oules, B., Debayle, D., Gay, A.S., Lacas-Gervais, S. et al. (2017) Localization and processing of the amyloid-beta protein precursor in mitochondria-associated membranes. J. Alzheimers Dis. 55, 1549–1570 10.3233/JAD-160953 27911326 PMC5181669

[16] Devi, L. and Ohno, M. (2012) Mitochondrial dysfunction and accumulation of the beta-secretase-cleaved C-terminal fragment of APP in Alzheimer's disease transgenic mice. Neurobiol Dis. 45, 417–424 10.1016/j.nbd.2011.09.001 21933711 PMC3225635

[17] Devi, L., Prabhu, B.M., Galati, D.F., Avadhani, N.G. and Anandatheerthavarada, H.K. (2006) Accumulation of amyloid precursor protein in the mitochondrial import channels of human Alzheimer's disease brain is associated with mitochondrial dysfunction. J Neurosci. 26, 9057–9068 10.1523/JNEUROSCI.1469-06.2006 16943564 PMC6675337

[18] Hansson Petersen, C.A., Alikhani, N., Behbahani, H., Wiehager, B., Pavlov, P.F., Alafuzoff, I. et al. (2008) The amyloid beta-peptide is imported into mitochondria via the TOM import machinery and localized to mitochondrial cristae. Proc. Natl Acad. Sci. U.S.A. 105, 13145–13150 10.1073/pnas.0806192105 18757748 PMC2527349

[19] Pavlov, P.F., Wiehager, B., Sakai, J., Frykman, S., Behbahani, H., Winblad, B. et al. (2011) Mitochondrial gamma-secretase participates in the metabolism of mitochondria-associated amyloid precursor protein. FASEB J. 25, 78–88 10.1096/fj.10-157230 20833873

[20] Pera, M., Larrea, D., Guardia-Laguarta, C., Montesinos, J., Velasco, K.R., Agrawal, R.R. et al. (2017) Increased localization of APP-C99 in mitochondria-associated ER membranes causes mitochondrial dysfunction in Alzheimer disease. EMBO J. 36, 3356–3371 10.15252/embj.201796797 29018038 PMC5731665

[21] Gabuzda, D., Busciglio, J., Chen, L.B., Matsudaira, P. and Yankner, B.A. (1994) Inhibition of energy metabolism alters the processing of amyloid precursor protein and induces a potentially amyloidogenic derivative. J. Biol. Chem. 269, 13623–8 10.1016/S0021-9258(17)36875-8 8175797

[22] Gasparini, L., Racchi, M., Benussi, L., Curti, D., Binetti, G., Bianchetti, A. et al. (1997) Effect of energy shortage and oxidative stress on amyloid precursor protein metabolism in COS cells. Neurosci. Lett. 231, 113–117 10.1016/s0304-3940(97)00536-3 9291153

[23] Henriques, A.G., Domingues, S.C., Fardilha, M., da Cruz e Silva, E.F. and da Cruz e Silva, O.A. (2005) Sodium azide and 2-deoxy-D-glucose-induced cellular stress affects phosphorylation-dependent AbetaPP processing. J. Alzheimers Dis. 7, 201–212 10.3233/jad-2005-7302 16006663

[24] Domingues, S.C., Henriques, A.G., Wu, W., da Cruz e Silva, E.F. and da Cruz e Silva, O.A. (2007) Altered subcellular distribution of the Alzheimer's amyloid precursor protein under stress conditions. Ann. N. Y. Acad. Sci. 1096, 184–195 10.1196/annals.1397.085 17405930

[25] Gasparini, L., Benussi, L., Bianchetti, A., Binetti, G., Curti, D., Govoni, S. et al. (1999) Energy metabolism inhibition impairs amyloid precursor protein secretion from Alzheimer's fibroblasts. Neurosci. Lett. 263, 197–200 10.1016/s0304-3940(99)00155-x 10213169

[26] Fu, W., Shi, D., Westaway, D. and Jhamandas, J.H. (2015) Bioenergetic mechanisms in astrocytes may contribute to amyloid plaque deposition and toxicity. J. Biol. Chem. 290, 12504–12513 10.1074/jbc.M114.618157 25814669 PMC4432272

[27] Mao, P., Manczak, M., Calkins, M.J., Truong, Q., Reddy, T.P., Reddy, A.P. et al. (2012) Mitochondria-targeted catalase reduces abnormal APP processing, amyloid beta production and BACE1 in a mouse model of Alzheimer'sAlzheimer's disease: implications for neuroprotection and lifespan extension. Hum. Mol. Genet. 21, 2973–2990 10.1093/hmg/dds128 22492996 PMC3373244

[28] Dumont, M., Wille, E., Stack, C., Calingasan, N.Y., Beal, M.F. and Lin, M.T. (2009) Reduction of oxidative stress, amyloid deposition, and memory deficit by manganese superoxide dismutase overexpression in a transgenic mouse model of Alzheimer's disease. FASEB J. 23, 2459–2466 10.1096/fj.09-132928 19346295 PMC2717785

[29] Fukui, H., Diaz, F., Garcia, S. and Moraes, C.T. (2007) Cytochrome c oxidase deficiency in neurons decreases both oxidative stress and amyloid formation in a mouse model of Alzheimer's disease. Proc. Natl Acad. Sci. U.S.A. 104, 14163–8 10.1073/pnas.0705738104 17715058 PMC1955773

[30] Troutwine, B.R., Strope, T.A., Franczak, E., Lysaker, C.R., Hamid, L., Mansel, C. et al. (2022) Mitochondrial function and abeta in Alzheimer's disease postmortem brain. Neurobiol. Dis. 171, 105781 10.1016/j.nbd.2022.105781 35667615 PMC9329272

[31] Bosetti, F., Brizzi, F., Barogi, S., Mancuso, M., Siciliano, G., Tendi, E.A. et al. (2002) Cytochrome c oxidase and mitochondrial F1F0-ATPase (ATP synthase) activities in platelets and brain from patients with Alzheimer's disease. Neurobiol. Aging 23, 371–376 10.1016/s0197-4580(01)00314-1 11959398

[32] Cardoso, S.M., Proenca, M.T., Santos, S., Santana, I. and Oliveira, C.R. (2004) Cytochrome c oxidase is decreased in Alzheimer's disease platelets. Neurobiol. Aging 25, 105–110 10.1016/s0197-4580(03)00033-2 14675736

[33] Parker, Jr, W.D. and Parks, J.K. (1995) Cytochrome c oxidase in Alzheimer's disease brain: purification and characterization. Neurology 45, 482–486 10.1212/wnl.45.3.482 7898701

[34] Wilkins, H.M., Koppel, S.J., Bothwell, R., Mahnken, J., Burns, J.M. and Swerdlow, R.H. (2017) Platelet cytochrome oxidase and citrate synthase activities in APOE epsilon4 carrier and non-carrier Alzheimer's disease patients. Redox Biol. 12, 828–832 10.1016/j.redox.2017.04.010 28448944 PMC5406545

[35] Leuner, K., Schutt, T., Kurz, C., Eckert, S.H., Schiller, C., Occhipinti, A. et al. (2012) Mitochondrion-derived reactive oxygen species lead to enhanced amyloid beta formation. Antioxid. Redox Signal. 16, 1421–1433 10.1089/ars.2011.4173 22229260 PMC3329950

[36] Stojakovic, A., Trushin, S., Sheu, A., Khalili, L., Chang, S.Y., Li, X. et al. (2021) Partial inhibition of mitochondrial complex I ameliorates Alzheimer's disease pathology and cognition in APP/PS1 female mice. Commun. Biol. 4, 61 10.1038/s42003-020-01584-y 33420340 PMC7794523

[37] Zhang, L., Zhang, S., Maezawa, I., Trushin, S., Minhas, P., Pinto, M. et al. (2015) Modulation of mitochondrial complex I activity averts cognitive decline in multiple animal models of familial Alzheimer's disease. EBioMedicine 2, 294–305 10.1016/j.ebiom.2015.03.009 26086035 PMC4465115

[38] Lu, X.Y., Huang, S., Chen, Q.B., Zhang, D., Li, W., Ao, R. et al. (2020) Metformin ameliorates abeta pathology by insulin-degrading enzyme in a transgenic mouse model of Alzheimer's disease. Oxid. Med. Cell. Longev. 2020, 2315106 10.1155/2020/2315106 32377293 PMC7191377

[39] Trifunovic, A., Wredenberg, A., Falkenberg, M., Spelbrink, J.N., Rovio, A.T., Bruder, C.E. et al. (2004) Premature ageing in mice expressing defective mitochondrial DNA polymerase. Nature 429, 417–423 10.1038/nature02517 15164064

[40] Scheffler, K., Krohn, M., Dunkelmann, T., Stenzel, J., Miroux, B., Ibrahim, S. et al. (2012) Mitochondrial DNA polymorphisms specifically modify cerebral beta-amyloid proteostasis. Acta Neuropathol. 124, 199–208 10.1007/s00401-012-0980-x 22526016 PMC3694593

[41] Pinto, M., Pickrell, A.M., Fukui, H. and Moraes, C.T. (2013) Mitochondrial DNA damage in a mouse model of Alzheimer's disease decreases amyloid beta plaque formation. Neurobiol. Aging 34, 2399–2407 10.1016/j.neurobiolaging.2013.04.014 23702344 PMC4020357

[42] Cardoso, S.M., Santana, I., Swerdlow, R.H. and Oliveira, C.R. (2004) Mitochondria dysfunction of Alzheimer's disease cybrids enhances abeta toxicity. J. Neurochem. 89, 1417–1426 10.1111/j.1471-4159.2004.02438.x 15189344

[43] Khan, S.M., Cassarino, D.S., Abramova, N.N., Keeney, P.M., Borland, M.K., Trimmer, P.A. et al. (2000) Alzheimer's disease cybrids replicate beta-amyloid abnormalities through cell death pathways. Ann. Neurol. 48, 148–155 10.1002/1531-8249(200008)48:2<148::AIDANA3>3.0.CO;2-7 10939564

[44] Silva, D.F., Selfridge, J.E., Lu J, E.L., Roy, N., Hutfles, L., Burns, J.M. et al. (2013) Bioenergetic flux, mitochondrial mass and mitochondrial morphology dynamics in AD and MCI cybrid cell lines. Hum. Mol. Genet. 22, 3931–3946 10.1093/hmg/ddt247 23740939 PMC3888119

[45] Swerdlow, R.H., Koppel, S., Weidling, I., Hayley, C., Ji, Y. and Wilkins, H.M. (2017) Mitochondria, cybrids, aging, and Alzheimer's disease. Prog. Mol. Biol. Transl. Sci. 146, 259–302 10.1016/bs.pmbts.2016.12.017 28253988 PMC5864124

[46] Trimmer, P.A., Keeney, P.M., Borland, M.K., Simon, F.A., Almeida, J., Swerdlow, R.H. et al. (2004) Mitochondrial abnormalities in cybrid cell models of sporadic Alzheimer's disease worsen with passage in culture. Neurobiol. Dis. 15, 29–39 10.1016/j.nbd.2003.09.011 14751768

[47] Wilkins, H.M., Troutwine, B.R., Menta, B.W., Manley, S.J., Strope, T.A., Lysaker, C.R. et al. (2022) Mitochondrial membrane potential influences amyloid-beta protein precursor localization and amyloid-beta secretion. J. Alzheimers Dis. 85, 381–394 10.3233/JAD-215280 34806611 PMC9212216

[48] Yang, T.T., Hsu, C.T. and Kuo, Y.M. (2009) Amyloid precursor protein, heat-shock proteins, and Bcl-2 form a complex in mitochondria and modulate mitochondria function and apoptosis in N2a cells. Mech. Ageing Dev. 130, 592–601 10.1016/j.mad.2009.07.002 19622370

[49] Area-Gomez, E., Del Carmen Lara Castillo, M., Tambini, M.D., Guardia-Laguarta, C., de Groof, A.J., Madra, M. et al. (2012) Upregulated function of mitochondria-associated ER membranes in Alzheimer disease. EMBO J. 31, 4106–4123 10.1038/emboj.2012.202 22892566 PMC3492725

[50] Lopez Sanchez, M.I.G., Waugh, H.S., Tsatsanis, A., Wong, B.X., Crowston, J.G., Duce, J.A. et al. (2017) Amyloid precursor protein drives down-regulation of mitochondrial oxidative phosphorylation independent of amyloid beta. Sci. Rep. 7, 9835 10.1038/s41598-017-10233-0 28852095 PMC5574989

[51] Keil, U., Bonert, A., Marques, C.A., Scherping, I., Weyermann, J., Strosznajder, J.B. et al. (2004) Amyloid beta-induced changes in nitric oxide production and mitochondrial activity lead to apoptosis. J. Biol. Chem. 279, 50310–50320 10.1074/jbc.M405600200 15371443

[52] Wang, Y., Wu, F., Pan, H., Zheng, W., Feng, C., Wang, Y. et al. (2016) Lost region in amyloid precursor protein (APP) through TALEN-mediated genome editing alters mitochondrial morphology. Sci. Rep. 6, 22244 10.1038/srep22244 26924205 PMC4770288

[53] Del Prete, D., Rice, R.C., Rajadhyaksha, A.M. and D'Adamio, L. (2016) Amyloid precursor protein (APP) may act as a substrate and a recognition unit for CRL4CRBN and Stub1 E3 ligases facilitating ubiquitination of proteins involved in presynaptic functions and neurodegeneration. J. Biol. Chem. 291, 17209–17227 10.1074/jbc.M116.733626 27325702 PMC5016122

[54] Schaefer, P.M., von Einem, B., Walther, P., Calzia, E. and von Arnim, C.A. (2016) Metabolic characterization of intact cells reveals intracellular amyloid beta but not its precursor protein to reduce mitochondrial respiration. PLoS ONE 11, e0168157 10.1371/journal.pone.0168157 28005987 PMC5178995

[55] Zheng, H., Jiang, M., Trumbauer, M.E., Sirinathsinghji, D.J., Hopkins, R., Smith, D.W. et al. (1995) beta-Amyloid precursor protein-deficient mice show reactive gliosis and decreased locomotor activity. Cell 81, 525–531 10.1016/0092-8674(95)90073-x 7758106

[56] Montagna, E., Crux, S., Luckner, M., Herber, J., Colombo, A.V., Marinkovic, P. et al. (2019) *In vivo* Ca^2+^ imaging of astrocytic microdomains reveals a critical role of the amyloid precursor protein for mitochondria. Glia 67, 985–998 10.1002/glia.23584 30667091

[57] Walls, K.C., Coskun, P., Gallegos-Perez, J.L., Zadourian, N., Freude, K., Rasool, S. et al. (2012) Swedish Alzheimer mutation induces mitochondrial dysfunction mediated by HSP60 mislocalization of amyloid precursor protein (APP) and beta-amyloid. J. Biol. Chem. 287, 30317–30327 10.1074/jbc.M112.365890 22753410 PMC3436283

[58] Vaillant-Beuchot, L., Mary, A., Pardossi-Piquard, R., Bourgeois, A., Lauritzen, I., Eysert, F. et al. (2021) Accumulation of amyloid precursor protein C-terminal fragments triggers mitochondrial structure, function, and mitophagy defects in Alzheimer's disease models and human brains. Acta Neuropathol. 141, 39–65 10.1007/s00401-020-02234-7 33079262 PMC7785558

[59] Cheng, G., Yu, Z., Zhou, D. and Mattson, M.P. (2002) Phosphatidylinositol-3-kinase-Akt kinase and p42/p44 mitogen-activated protein kinases mediate neurotrophic and excitoprotective actions of a secreted form of amyloid precursor protein. Exp. Neurol. 175, 407–414 10.1006/exnr.2002.7920 12061870

[60] Hamilton, D.L., Findlay, J.A., Montagut, G., Meakin, P.J., Bestow, D., Jalicy, S.M. et al. (2014) Altered amyloid precursor protein processing regulates glucose uptake and oxidation in cultured rodent myotubes. Diabetologia 57, 1684–1692 10.1007/s00125-014-3269-x 24849570 PMC4079947

[61] Cimdins, K., Waugh, H.S., Chrysostomou, V., Lopez Sanchez, M.I.G., Johannsen, V.A., Cook, M.J. et al. (2019) Amyloid precursor protein mediates neuronal protection from rotenone toxicity. Mol. Neurobiol. 56, 5471–5482 10.1007/s12035-018-1460-7 30612335 PMC6614131

[62] Dar, N.J. and Glazner, G.W. (2020) Deciphering the neuroprotective and neurogenic potential of soluble amyloid precursor protein alpha (sAPPalpha). Cell. Mol. Life Sci. 77, 2315–2330 10.1007/s00018-019-03404-x 31960113 PMC11105086

[63] Ward, M.W., Concannon, C.G., Whyte, J., Walsh, C.M., Corley, B. and Prehn, J.H. (2010) The amyloid precursor protein intracellular domain(AICD) disrupts actin dynamics and mitochondrial bioenergetics. J. Neurochem. 113, 275–284 10.1111/j.1471-4159.2010.06615.x 20405578

[64] Goiran, T., Duplan, E., Chami, M., Bourgeois, A., El Manaa, W., Rouland, L. et al. (2018) beta-amyloid precursor protein intracellular domain controls mitochondrial function by modulating phosphatase and tensin homolog-induced kinase 1 transcription in cells and in Alzheimer mice models. Biol. Psychiatry 83, 416–427 10.1016/j.biopsych.2017.04.011 28587718

[65] Shang, Y., Sun, X., Chen, X., Wang, Q., Wang, E.J., Miller, E. et al. (2022) A CHCHD6-APP axis connects amyloid and mitochondrial pathology in Alzheimer's disease. Acta Neuropathol. 144, 911–938 10.1007/s00401-022-02499-0 36104602 PMC9547808

[66] Sandberg, A.A., Manning, E., Wilkins, H.M., Mazzarino, R., Minckley, T., Swerdlow, R.H. et al. (2022) Mitochondrial targeting of amyloid-beta protein precursor intracellular domain induces hippocampal cell death via a mechanism distinct from amyloid-beta. J. Alzheimers Dis. 86, 1727–1744 10.3233/JAD-215108 35253745 PMC10084495

[67] Hansson, C.A., Frykman, S., Farmery, M.R., Tjernberg, L.O., Nilsberth, C., Pursglove, S.E. et al. (2004) Nicastrin, presenilin, APH-1, and PEN-2 form active gamma-secretase complexes in mitochondria. J. Biol. Chem. 279, 51654–51660 10.1074/jbc.M404500200 15456764

[68] Francelin, C., Mitter, S.K., Qian, Q., Barodia, S.K., Ip, C., Qi, X. et al. (2021) BACE1 inhibition increases susceptibility to oxidative stress by promoting mitochondrial damage. Antioxidants (Basel) 10, 1539 10.3390/antiox10101539 34679674 PMC8532805

[69] Falkevall, A., Alikhani, N., Bhushan, S., Pavlov, P.F., Busch, K., Johnson, K.A. et al. (2006) Degradation of the amyloid beta-protein by the novel mitochondrial peptidasome, PreP. J. Biol. Chem. 281, 29096–29104 10.1074/jbc.M602532200 16849325

[70] Mossmann, D., Vogtle, F.N., Taskin, A.A., Teixeira, P.F., Ring, J., Burkhart, J.M. et al. (2014) Amyloid-beta peptide induces mitochondrial dysfunction by inhibition of preprotein maturation. Cell Metab. 20, 662–669 10.1016/j.cmet.2014.07.024 25176146

[71] Crouch, P.J., Blake, R., Duce, J.A., Ciccotosto, G.D., Li, Q.X., Barnham, K.J. et al. (2005) Copper-dependent inhibition of human cytochrome c oxidase by a dimeric conformer of amyloid-beta1-42. J. Neurosci. 25, 672–679 10.1523/JNEUROSCI.4276-04.2005 15659604 PMC6725334

[72] Caspersen, C., Wang, N., Yao, J., Sosunov, A., Chen, X., Lustbader, J.W. et al. (2005) Mitochondrial Abeta: a potential focal point for neuronal metabolic dysfunction in Alzheimer's disease. FASEB J. 19, 2040–2041 10.1096/fj.05-3735fje 16210396

[73] Lustbader, J.W., Cirilli, M., Lin, C., Xu, H.W., Takuma, K., Wang, N. et al. (2004) ABAD directly links Abeta to mitochondrial toxicity in Alzheimer's disease. Science 304, 448–452 10.1126/science.1091230 15087549

[74] Takuma, K., Yao, J., Huang, J., Xu, H., Chen, X., Luddy, J. et al. (2005) ABAD enhances Abeta-induced cell stress via mitochondrial dysfunction. FASEB J. 19, 597–598 10.1096/fj.04-2582fje 15665036

[75] de la Cueva, M., Antequera, D., Ordonez-Gutierrez, L., Wandosell, F., Camins, A., Carro, E. et al. (2022) Amyloid-beta impairs mitochondrial dynamics and autophagy in Alzheimer's disease experimental models. Sci. Rep. 12, 10092 10.1038/s41598-022-13683-3 35710783 PMC9203760

[76] Manczak, M., Anekonda, T.S., Henson, E., Park, B.S., Quinn, J. and Reddy, P.H. (2006) Mitochondria are a direct site of A beta accumulation in Alzheimer's disease neurons: implications for free radical generation and oxidative damage in disease progression. Hum. Mol. Genet. 15, 1437–1449 10.1093/hmg/ddl066 16551656

[77] Wang, X., Su, B., Siedlak, S.L., Moreira, P.I., Fujioka, H., Wang, Y. et al. (2008) Amyloid-beta overproduction causes abnormal mitochondrial dynamics via differential modulation of mitochondrial fission/fusion proteins. Proc. Natl Acad. Sci. U.S.A. 105, 19318–19323 10.1073/pnas.0804871105 19050078 PMC2614759

[78] Manczak, M., Calkins, M.J. and Reddy, P.H. (2011) Impaired mitochondrial dynamics and abnormal interaction of amyloid beta with mitochondrial protein Drp1 in neurons from patients with Alzheimer's disease: implications for neuronal damage. Hum. Mol. Genet. 20, 2495–2509 10.1093/hmg/ddr139 21459773 PMC3109997

[79] Calvo-Rodriguez, M., Hernando-Perez, E., Nunez, L. and Villalobos, C. (2019) Amyloid beta oligomers increase ER-mitochondria Ca^2+^ cross talk in young hippocampal neurons and exacerbate aging-induced intracellular Ca^2+^ remodeling. Front. Cell Neurosci. 13, 22 10.3389/fncel.2019.00022 30800057 PMC6376150

[80] Area-Gomez, E., de Groof, A.J., Boldogh, I., Bird, T.D., Gibson, G.E., Koehler, C.M. et al. (2009) Presenilins are enriched in endoplasmic reticulum membranes associated with mitochondria. Am. J. Pathol. 175, 1810–1816 10.2353/ajpath.2009.090219 19834068 PMC2774047

[81] Hedskog, L., Pinho, C.M., Filadi, R., Ronnback, A., Hertwig, L., Wiehager, B. et al. (2013) Modulation of the endoplasmic reticulum-mitochondria interface in Alzheimer's disease and related models. Proc. Natl Acad. Sci. U.S.A. 110, 7916–7921 10.1073/pnas.1300677110 23620518 PMC3651455

